# Influence of Seismic Loading on Segment Opening of a Shield Tunnel

**DOI:** 10.1155/2014/387210

**Published:** 2014-04-03

**Authors:** Yang Chun-shan, Mo Hai-hong, Chen Jun-sheng, Wang Yi-zhao

**Affiliations:** ^1^School of Civil Engineering and Transportation, South China University of Technology, Guangzhou 510641, China; ^2^State Key Laboratory of Subtropical Building Science, Guangzhou 510641, China

## Abstract

The influence of seismic loading on segment opening of a shield tunnel was explored using the dynamic finite element method to analyze the distribution of segment opening under multidirectional seismic loading, combined with a typical engineering installation. The calculation of segment opening was deduced from equivalent continuous theory and segment opening was obtained through calculations. The results show that the scope of influence of the foundation excavation on segment opening is mainly resigned to within 5 segment rings next to the diaphragm wall and 4 joints nearest the working well when the tunnel is first excavated followed by the working well in the excavation order. The effect of seismic loading on segment opening is significant, and the minimum increase of the maximal segment opening owing to seismic loading is 16%, while that of the average opening is 27%. Segment opening under bidirectional coupled seismic loading is significantly greater than that under one-dimensional seismic loading. On the basis of the numerical calculations, the seismic acceleration and segment opening caused by seismic action were normalized, and a new calculation method was proposed for predicting the maximal segment opening of a shield tunnel at different depths under conditions of seismic loading.

## 1. Introduction

Subway shield tunneling constitutes a major segment of the civil infrastructure that serves public transportation [1, 2]. Damage to shield tunnels resulting from seismic loading was first observed during the Niigata earthquake and then, later, during the Alaska earthquake. During the Kobe earthquake, the majority of devastation occurred in several urban subway shield tunnels [3]. The earthquake in Taiwan is another example of severe subway structure damage [4]. As a result, the effect of earthquakes on subway shield tunnels became an important issue [5], and several studies involving seismic response analysis of shield tunneling were carried out.

In recent years, researchers have used a variety of approaches, including numerical and analytical methods as well as experimental studies, to explore the seismic response of shield tunnels. Argyroudis et al. [6–9] used numerical methods for this purpose. Hashash and Zhao [10, 11] used an analytical method to explore the effect of earthquakes on tunnel structures and applied it to tunnel design. Hildyard et al. [12–14] conducted experimental studies of the response characteristics and mechanisms of earthquake damage to utility tunnels.

However, most of these studies considered only the lateral shear wave of an earthquake and tended to focus on the mechanical characteristics of the lining structure, while studies on seismic response from the standpoint of multidirectional earthquake action and segment opening caused by seismic loading are much less common. Investigations of actual seismic damage have shown that an earthquake is much more devastating when the seismic wave has a larger acceleration in the vertical direction (such as the case in the Kobe earthquake). As such, the damage observed at tunnel entrances and segment joints deviates from the standard structural response, resulting in substantial relative separation between segment rings and segment circumferential opening, which induces leakage and other conditions. Therefore, it is quite necessary to investigate circumferential opening of the lining segments under multidirectional seismic loading.

At present, equivalent continuous theory developed by Yukio et al. [15, 16], which has been used to calculate segment opening under static loading conditions in the absence of seismic action, can be adopted to calculate circumferential opening of shield tunnels. Based on this, the shield tunnel of the Guangzhou Xinjiang water diversion project was taken as an example and the dynamic finite element method was used to analyze the seismic response of segment opening under various seismic activities. A numerical analysis method was conducted by commercial FE software to couple seismic effects to equivalent continuous theory, so that segment opening caused by seismic loading could be more accurately predicted, and to provide a reference for seismic design of shield tunnels.

## 2. Finite Element Modelling

The Guangzhou Xinjiang water diversion project is a crucial lifeline engineering project for improving water quality in Guangzhou. A nonexcavating shield tunneling method was adopted to cross the major transportation hub in the urban area. The construction exhibits substantial changes in structural stiffness from tunnel to working well, so the scope of analysis in this work consists of the receiving well and the segment of the tunnel which lies next to the well. The tunnel was first excavated followed by the working well in the excavation order. The receiving well is a rectangular foundation pit, and its clearance length, width, and depth are 28 m, 14 m, and 22.6 m, respectively. Support for the foundation pit consists of a 1.2 m diaphragm wall and 4 reinforced concrete internal bracings. The embedded depth of the diaphragm wall is about 32 m. The outer diameter of the lining segment is 6 m and inner diameter is 5.4 m. The lining consists of 6 segments.

**Figure 1:**
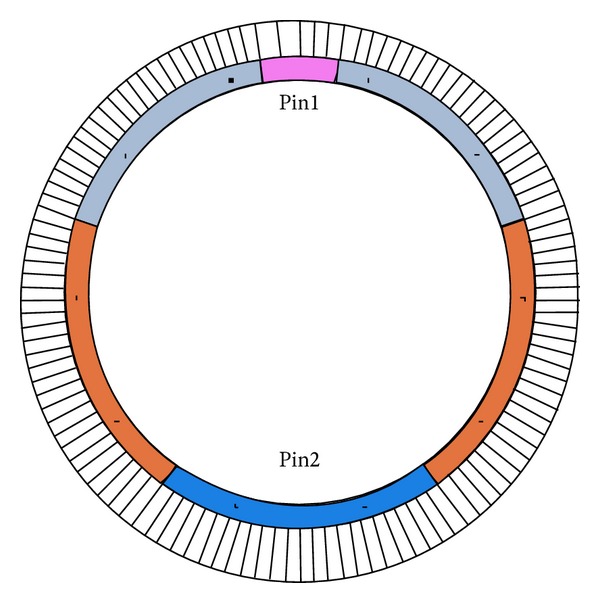
Grouting pressure acting on the segments.

According to the geological survey report and related structural design of the project, the parameters of the soil and the structure are listed in Tables 1, 2, and 3. The boundary of the model is set at 2 or 3 times the tunnel diameter [17]; the geometrical length (the dimension of *x*-axis), width (*y*-axis), and height (*z*-axis) of the model are 90 m, 60 m, and 60 m, respectively. Empirically, the equivalent Young's modulus of a straight bolt is 5.4 × 10^7^ kPa and the segment circular seam bears a 1600 kN/m pressure along the circumferential direction in the construction stage. In the present work, jacking forces were simplified to the pressure acting on a circular pad, and the equivalent pressure is 5400 kPa [18]. The grouting pressure Pin is between 0.1 MPa and 0.5 MPa. In order to reflect general conditions, Pin1 and Pin2, as shown in Figure 1, were assumed to be 0.30 MPa and 0.45 MPa, respectively [19]. According to the soil conditions, the excavation face pressure is 120 kPa.

The artificial boundary of the model adopted the viscous boundary. In order to allow for energy exchange between the limited soil of the model and the infinite soil external to the model and to satisfy the condition that waves radiate through the virtual boundary to infinity, a damper was applied at the boundary using a viscous damping force which is proportional to the velocity. For this purpose, we employed Rayleigh damping [6, 20], as given by

(1) $$\begin{array}{c} \left[C\right]=\alpha \left[M\right]+\beta \left[K\right], \end{array}$$

wherein [*C*] is the damping matrix, [*M*] is the mass matrix, [*K*] is the stiffness matrix, *α* is the quality damping coefficient, and *β* is the stiffness damping coefficient. According to the vibration mode decomposition method, *α* and *β* can be expressed by two modal damping ratios and the corresponding natural frequency of vibration, as given by

(2) $$\begin{array}{c} \alpha =\frac{2\left(\left(\xi_{i}/\omega_{i}\right)-\left(\xi_{j}/\omega_{j}\right)\right)}{\left(\left(1/\omega_{i}^{2}\right)-\left(1/\omega_{j}^{2}\right)\right)},\\ \beta =\frac{2\left(\xi_{j}\omega_{j}-\xi_{i}\omega_{i}\right)}{\left(\omega_{j}^{2}-\omega_{i}^{2}\right)}, \end{array}$$

wherein *ξ*_*i*_ and *ω*_*i*_ are, respectively, the damping ratio and natural frequency of vibration for mode *i*. For this analysis, *ξ* is 0.05, and *α* and *β* were calculated from the natural frequencies of the two main vibrational modes which have the largest periods.

**Figure 2:**
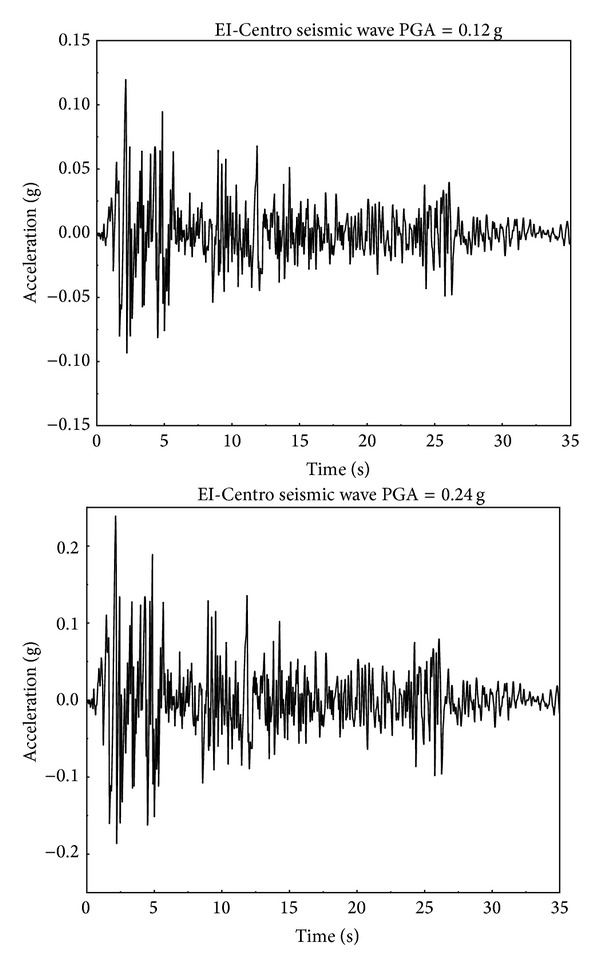
Time-history curves of ground motion acceleration for two different seismic wave amplitudes.

The actual acceleration records for the strong earthquake (>intensity VII) have not yet been obtained. Therefore, the existing seismic wave records for a strong earthquake that are consistent with the actual site conditions were selected. The project resides on a type II site which is suitable for medium hardness soil. Therefore, the EI-Centro seismic wave (270° direction), which suits a type II site, was selected for this study. This seismic wave tends to stabilize within 35 s, and, therefore, seismic wave duration of 35 s was adopted. The seismic amplitude is determined by the earthquake intensity, for which two have been selected. These include an earthquake intensity VII, which has a probability of occurrence exceeding 10% in 50 years and a PGA (peak ground acceleration) of 0.12 g, and an earthquake intensity III, which has a probability of occurrence exceeding 2% in 100 years and a PGA of 0.24 g [11–21]. The time-history curves of seismic wave acceleration for these two earthquake amplitudes are shown in Figure 2.

**Figure 3:**
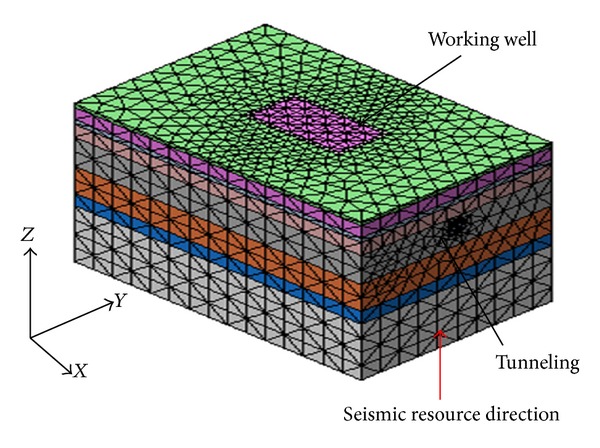
A representation of the general numerical modelling scheme employed.

**Figure 4:**
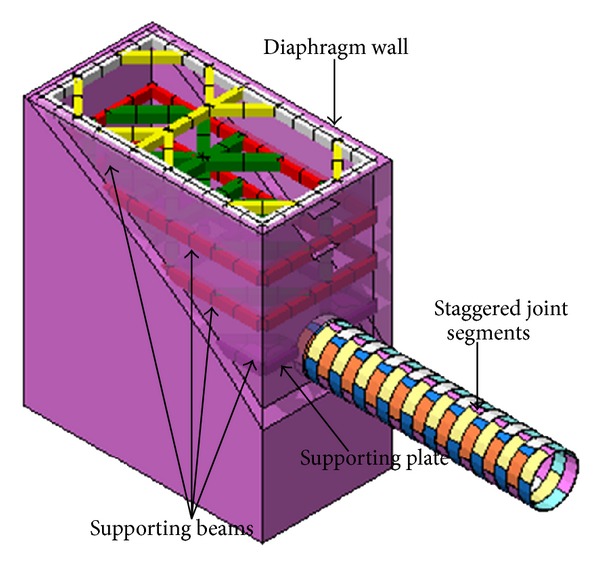
Schematic representation of the model employed for the supporting structure and segments.

Three-dimensional solid elements were applied to simulate soil, segments, segment connections, grouting, and the diaphragm wall of the model, and beam elements were used to simulate supporting structures. The supporting plate and shield were stimulated with shell elements. Goodman [22] contact elements were applied to analyze relative sliding between soil, tunnel, and diaphragm wall. The soil and tunnel structures were modelled using the Mohr-Coulomb model and the elastic model, respectively. A representation of the general numerical modelling scheme employed is shown in Figure 3. The supporting structures and tunnel were modelled as shown in Figure 4. The model includes 20 segment rings numbered from 1 to 20 with the ring nearest the foundation pit set as number 20. The 1st segment resides a certain distance away from the working well, and it is slightly affected by the foundation excavation. Therefore, the boundary of the 1st segment includes lateral and vertical direction displacement constraints.

## 3. Results and Analysis

In order to explore the effects of seismic loading along different directions on segment opening, four calculation conditions were analyzed as follows: (1) no seismic loading (static loading), (2) horizontal seismic loading (S-wave), (3) vertical seismic loading (P-wave), and (4) bidirectional coupled seismic loading.

There are 19 joints corresponding to the 20 segments and they are numbered from 1 to 19. According to the horizontal displacement of the segment joints, the relative displacement of segments was calculated, which represents segment opening. The segments are relatively open when segment openings are positive, while they are relatively compressed when openings are negative.

**Figure 5:**
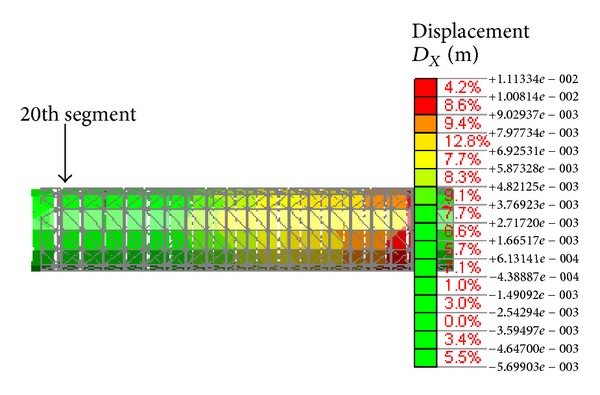
Displacement nephogram of the segments.

**Figure 6:**
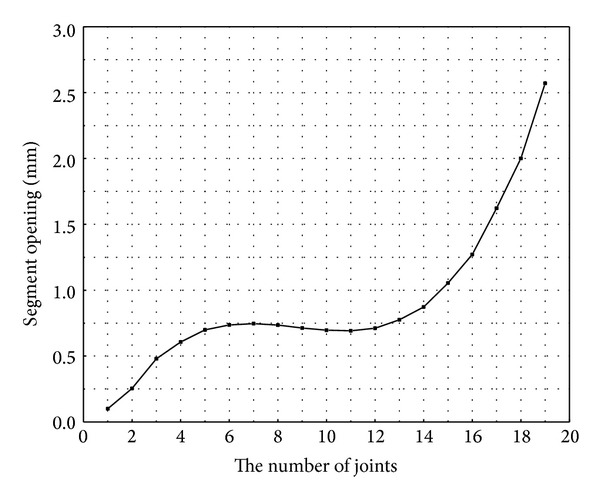
A graph of segment opening relative to joint number.

The displacement nephogram of the lining segments is shown in Figure 5 under static loading. The extent of segment opening is shown in Figure 6. Segment opening tends to increase from the 1st joint to the 19th joint. The maximal opening is 2.57 mm, which appears at the 19th joint. The maximal opening is greater than the joint elastic limit (1.11 mm) [23] and is less than the control value set by Guangzhou metro (3 mm) [24], indicating that the segment experiences no leakage. Figure 6 also indicates that segment opening significantly increases from the 16th joint upwards, and the latter joints begin to experience plastic deformation, such that the joints are in an elastic state before the 16th joint. This indicates that the scope of influence of the foundation excavation on segment opening is mainly resigned to the five segment rings near the diaphragm wall and the four joints next to the working well when the tunnel is first excavated followed by the working well in the excavation order.

**Figure 7:**
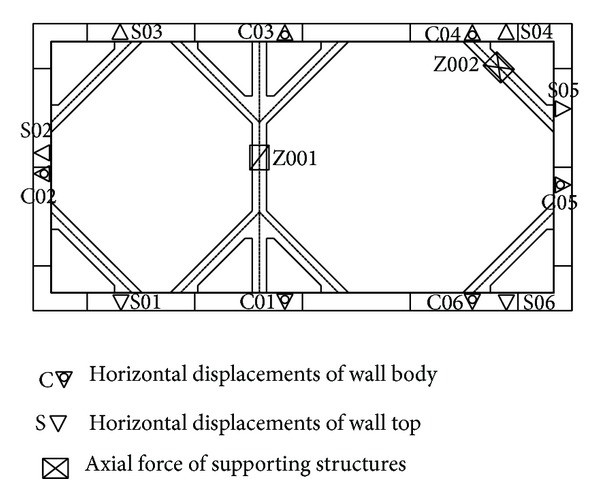
The distribution of monitoring points for the field measurements of displacements of the wall body and wall top as well as the internal force of the supporting structures.

**Figure 8:**
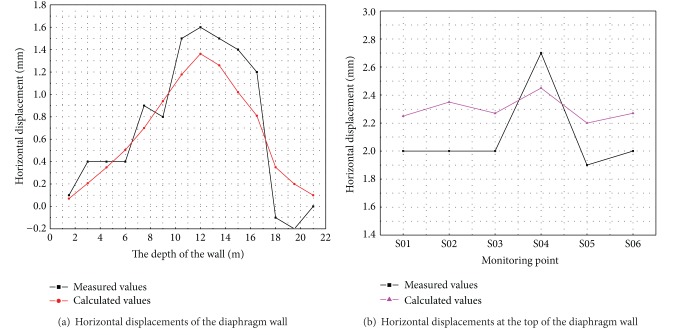
The comparison between measurements and simulation results.

Existing field measurements for the actual installation include displacements of the wall body and wall top as well as the internal force of the supporting structures. The monitoring points are shown in Figure 7. Comparison of the measured displacements of wall body (point C03) and wall top with calculation results will be used to verify the soundness of the model. The comparison between measured and simulation results is shown in Figure 8.

The comparison shows that the calculation results match the measured values obtained by author's field measurements well indicating that the numerical results reasonably reflect the displacement tendencies of the diaphragm wall. The modelling method is therefore effective in this work.

Horizontal seismic action denotes seismic response of the tunnel structure to a shear wave (S-wave), while vertical seismic action denotes seismic response of the tunnel structure to a compression wave (P-wave). We consider the effect of S-wave and P-wave and the bidirectional coupled effect of seismic action on segment opening. The vertical seismic acceleration is 0.65 times that of the horizontal seismic acceleration [25, 26]. The seismic source is perpendicular to the axial plane of the tunnel, as shown in Figure 3.

**Figure 9:**
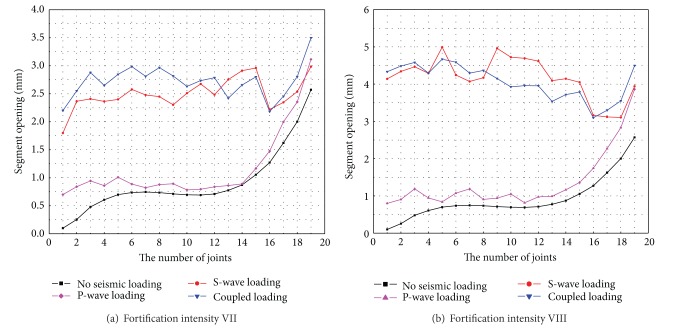
Segment opening under various seismic loading conditions.

Segment opening under various conditions of seismic loading is shown in Figure 9 and overlays the values of segment opening obtained under conditions of no seismic loading. The maximum and average values of segment opening and the joint deformation state for all seismic loading conditions are listed in Table 4.

Figure 9 indicates that segment opening behaviour is similar under medium seismic loading (equivalent to fortification intensity VII) and strong seismic loading (equivalent to fortification intensity VIII) conditions. The main seismic response of the tunnel is axial tension and compression under S-wave action. Meanwhile, segment opening increases and decreases under the effect of continuous tension and compression. The seismic responses of the tunnel are mainly bending and uplift under P-wave action, and segment opening presents an obviously increasing tendency from border to diaphragm wall.

Figure 9 and Table 4 show that seismic action has a significant influence on segment opening, where the minimum amplitude increases of the maximum and average values are 15% and 28%, respectively, relative to no seismic loading. All segment joints are in a state of plastic deformation and values of partial segment opening exceed the control value under medium earthquake conditions, while all values of segment opening exceed the control value under strong earthquake action when considering bidirectional coupled loading. Segment opening caused by bidirectional coupled loading is greater than that caused by unidirectional actions. The effect of P-wave seismic loading on segment opening is less than that of S-wave action, while it still has a major influence on segment opening, and, corresponding to fortification intensity VII and fortification intensity VIII, segment opening increased by 27.5% and 52.7% relative to that with no seismic loading, respectively. Therefore, in order to accurately and comprehensively study the seismic response of a tunnel, multidirectional seismic loading should be fully considered during the design phase of a shield tunnel.

## 4. Prediction Method of the Maximal Segment Opening under Seismic Loading

The calculation results shown in Figure 9 and listed in Table 4 indicate that the maximal segment opening is similar under different conditions for the same earthquake intensity. Therefore, the maximal segment opening caused by a P-wave action can, to some extent, represent the maximum caused by any other seismic loading and it can forecast the maximal opening under P-wave action to obtain the opening under seismic loading.

**Figure 10:**
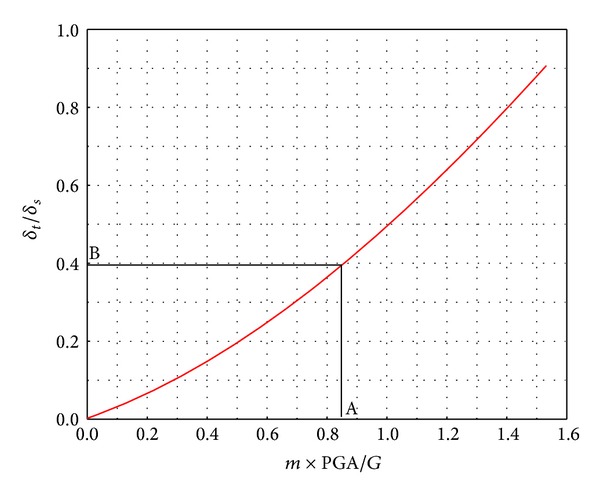
Modified calculating chart of segment opening owing to seismic loading.

In order to study the effects of earthquakes on segment opening and accurately predict the maximum segment opening under a given seismic loading, we used the static calculation function of segment opening, which is derived from equivalent continuous theory and law between the seismic acceleration and segment opening calculated by finite element method under P-wave loading. The inertial force of the tunnel caused by seismic action and the force due to gravity of the overlying soil were used to normalize seismic acceleration, and segment opening caused by static loading was taken as a standard to normalize segment opening caused by seismic loading. According to the law between the seismic acceleration and segment opening under P-wave loading, as shown in Figure 10, a correction calculation chart of seismic loading was proposed for predicting the maximal segment opening of a shield tunnel at different depths. We define *δ*_*t*_ and *δ*_*s*_ as the segment openings under seismic and static loading conditions, respectively. We assign *m* as the quality of a segment and *G* is the force due to gravity of the overlying soil in the range corresponding to the width of a segment ring.

The proposed steps for calculation of segment opening of a shield tunnel are as follows.

1. The calculation function of the maximal segment opening was deduced from equivalent continuous theory under static loading.
2. According to the seismic acceleration and the value of *G*, we locate the point A in Figure 10.
3. The corresponding segment opening ratio B can be found from point A.
4. The maximal segment opening caused by static loading is multiplied by the proportionality coefficient B to obtain the segment opening caused by seismic loading.
5. Segment opening under seismic loading conditions is thereby obtained.

**Figure 11:**
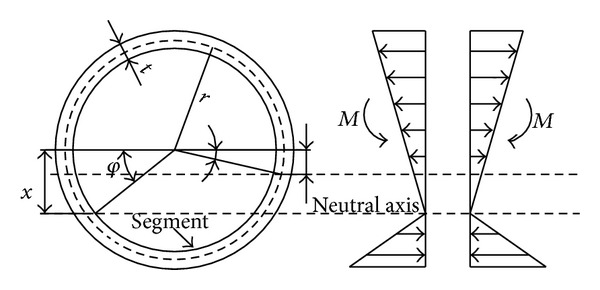
The longitudinal bending calculation chart.

According to the main ideas of this work, the maximal opening of the shield tunnel of the Guangzhou Xinjiang water diversion project was calculated under conditions of seismic loading. The seismic responses of the tunnel are mainly bending and uplift under P-wave action based on the previous analysis. Therefore, by the equivalent continuous theory, the position of the neutral axis (Figure 11) of a segment can meet (3) based on deformation coordination and force balance under static loading [25, 27]. Consider

(3) $$\begin{array}{c} \text{cot}\varphi +\varphi =\pi \left(\frac{1}{2}+\frac{k_{j1}l_{s}}{E_{c}A_{c}}\right), \end{array}$$

(4) $$\begin{array}{c} k_{j1}=\frac{nE_{j}A_{c}}{l}. \end{array}$$

The maximal opening can be deduced based on mechanical knowledge as follows:

(5) $$\begin{array}{c} \delta_{\max}=\frac{M_{\max}l_{s}}{E_{c}I_{c}}\frac{\pi \sin\varphi }{\cos^{3}\varphi }\left(r+x\right), \end{array}$$

wherein *k*_*j*1_ is the elastic stiffness of the longitudinal joints, *n* is the number of longitudinal bolts, *E*_*j*_ is the elastic modulus of bolts, *E*_*c*_ is the elastic modulus of the segment (kPa), *A*_*c*_ is the sectional area of segments (m^2^), *M*_max⁡_ is the maximum of the longitudinal bending moment (kN · m), *l*_*s*_ is the length of a segment ring (m), *I*_*c*_ is the inertial moment of the section of a segment (m^4^), *r* is the central radius of the segment (m), and *φ* and *x*, respectively, indicate the position and angle of the neutral axis when segment is curved and *x* = *r*sin⁡*φ*.

A beam-spring model was used to set up a longitudinal calculation model to calculate the maximal moment based on elastic foundation beams. Beam elements were used to simulate the tunnel. The coefficient of subgrade reaction is 14.1 MPa/m. The maximal bending moment calculated was 5.1113 × 10^3^ kN·m by calculating.

According to Table 3 and (4), the elastic stiffness (*k*_*j*1_) of a single bolt is 192284.7 kN/m, and this value is substituted into (3) to obtain the neutral axis location at an angle *φ* which is 1.15 rad. This value is then substituted into (5) to obtain a segment opening of 2.71 mm, which coincides with the numerical result under static loading.

The calculation of fortification intensity VII has a PGA value of 0.12 g, and the opening ratio B corresponding to Figure 10 is 0.387. Therefore, the maximal segment opening caused by an earthquake is 2.71 × 0.387 = 1.05 mm, and the maximal segment opening is 2.71 + 1.05 = 3.76 mm under the combined action of static and seismic loading. The result can be seen to coincide with the numerical value, which is 3.5 mm, obtained in this work. The maximal segment opening under conditions of seismic loading is 1.387 times that with no loading. This shows that it is quite necessary to consider the effects of an earthquake during the calculation of segment opening under seismic loading. By the same method, the maximal opening under fortification intensity VIII is 4.83 mm, which coincides with the numerical results of 4.46 mm. Therefore, the modified calculation method of the maximal segment opening proposed in this work is effective for conditions of seismic loading.

## 5. Conclusions

1. The scope of influence of the foundation pit excavation on segment opening is mainly within five segment rings adjacent to the diaphragm wall and the four joints nearest the working well with no seismic action. Therefore, local reinforcement can be considered in this range.
2. The effect of earthquakes on segment opening is significant. Under conditions of bidirectional coupled loading, all segment joints are in a state of plastic deformation and partial openings exceed the control value for a medium earthquake, while all openings exceed the control value for a strong earthquake.
3. Segment opening caused by bidirectional coupled seismic loading is significantly different from one-dimensional seismic loading. The former opening is greater than the latter. Therefore, the superimposition of two unidirectional earthquake waves should be fully considered.
4. A modified calculation method for predicting the maximal segment opening of a shield tunnel at different depths was proposed. The method can accurately predict the maximal segment opening under seismic loading.

## Figures and Tables

**Table 1:** Physicomechanical parameters of the soil.

Geotechnical name	Density (kg/m^3^)	Consolidated quick shear		Compression modulus (MPa)	Deformation modulus *E*_0_ (MPa)	Lateral pressure coefficient	Poisson ratio	Thickness of the soil (m)
		Cohesion (Pa)	Internal friction angle (°)					
Artificial filled Soil	1.90 × 10^3^	10.0 × 10^3^	18.0	4.7	10	0.49	0.33	1.5
Silty clay	1.99 × 10^3^	19.2 × 10^3^	15.2	5.86	12	0.43	0.30	4.5
Mud fine sand	2.00 × 10^3^	5.0 × 10^3^	25.0	5.93	18	0.39	0.28	1.9
Mud medium fine sand	2.01 × 10^3^	0.0	30.0	6.52	20	0.35	0.26	5.2
Mud fine sand	1.90 × 10^3^	4.0 × 10^3^	29.6	6.89	20	0.39	0.28	10.7
Mud medium coarse sand	1.98 × 10^3^	0.0	32.0	7.40	25	0.35	0.26	10.2
Mud sand gravel	1.90 × 10^3^	—	36.0	8.14	30	0.30	0.23	4.8
Strong weathered tuff	2.00 × 10^3^	—	—	—	120	—	—	21.2

**Table 2:** Parameters of the supporting structure.

Type	Density (kg/m^3^)	Length × width (mm × mm)	Thickness (mm)
Inner support 1	2.5 × 10^3^	800 × 1000	
Inner support 2, 3	2.5 × 10^3^	1000 × 1200	
Inner support 4	2.5 × 10^3^	800 × 1000	
Ring beam 1	2.5 × 10^3^	1000 × 1200	
Ring beam 2, 3	2.5 × 10^3^	1200 × 1200	
Ring beam 4	2.5 × 10^3^	1000 × 1000	
Support plate	2.5 × 10^3^		1000
Diaphragm wall	2.5 × 10^3^		1200
Base plate	2.5 × 10^3^		800

Attention the concrete grade of the continuous wall and inner structures is C30.

**Table 3:** Structural parameters of lining segments.

External diameter (m)	Inner diameter (m)	Ring width *l*_*s*_ (m)	Elastic modulus of concrete *E*_*c*_ (kPa)	Length of bolts (m)	Number of bolts	Elastic modulus of bolts *E*_*j*_ (kPa)
6	5.4	1.6	3.45 × 10^7^	0.4	11	2.06 × 10^8^

**Table 4:** Segment opening and joint deformation under different seismic loading conditions.

Conditions	Maximal opening (mm)	Average opening (mm)	Maximal opening by seismic action (mm)	Average opening by seismic action (mm)	Joint deformation state
No seismic loading	2.57	0.91	—	—	Partial plastic
S-wave of fortification intensity VII	2.98	2.51	0.41	1.6	Overall plastic
P-wave of fortification intensity VII	3.11	1.16	0.54	0.25	Partial plastic
Bidirectional coupled loading of fortification intensity VII	3.5	2.71	0.93	1.8	Overall plastic
S-wave of fortification intensity VIII	4.01	3.99	1.44	3.08	Overall plastic
P-wave of fortification intensity VIII	3.86	1.39	1.29	0.48	Partial plastic
Bidirectional coupled loading of fortification intensity VIII	4.49	4.30	1.92	3.39	Overall plastic
